# Supplementary material for: Identification of photoperiod-induced specific miRNAs in the adrenal glands of Sunite sheep (Ovis aries)
Source: Front Vet Sci. 2022 Jul 22;9:888207. doi: 10.3389/fvets.2022.888207 (PMC9354845; doi:10.3389/fvets.2022.888207)
Supplement: Supplementary file 1 [file Data_Sheet_1.ZIP › Supplementary file/miRDeep2 file.zip!/miRDeep2 file/Novel_Structure/LP42A3/Novel_9.pdf]

Provisional ID : NC_019480.2_22415
Score total : 1.2
Score for star read(s) : -1.3
Score for read counts : 0
Score for mfe : 0.9
Score for randfold : 1.6
Score for cons. seed :
Total read count : 153
Mature read count : 153
Loop read count : 0
Star read count : 0

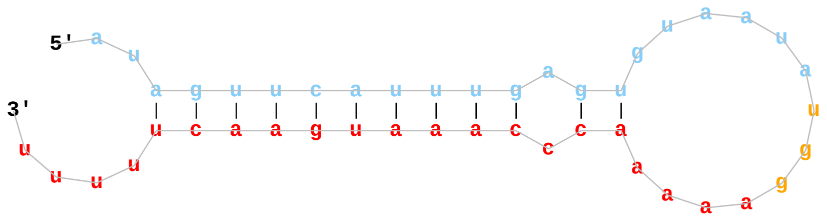


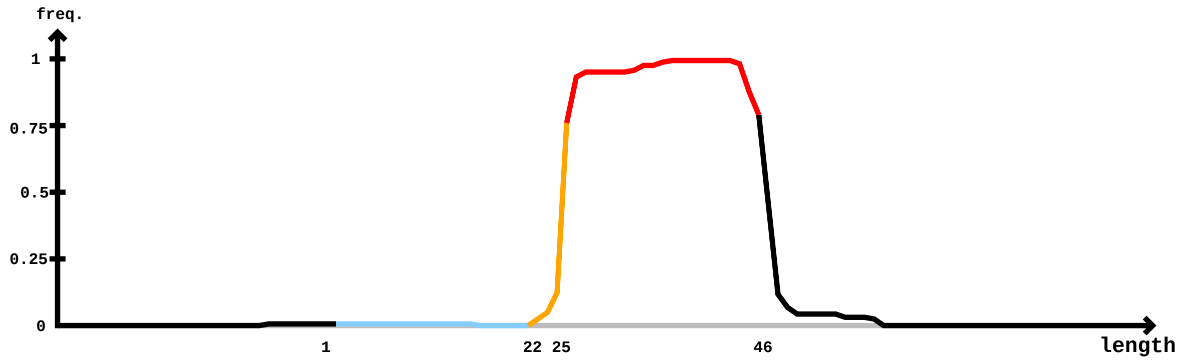


```
                                        Star                    Mature
5'- uuuuguaagguauauugaauuggcccaauaguucauuugaguguaaauauggaaaaacccaaaugaacuuuuuggccaacccaguacauuaccuacucauaccccacagucaug   -3'   exp
    ....(((.(((((((((..((((((.((.((((((((((.((..............)).)))))))))).)).))))))..)))))...))))))).................. reads mm sample
    ....................uggcccaauaguucauuugagu........................................................................ 1 0 seq
    .................................................uggaaaaGcccaaaugaacuu............................................ 1 1 seq
    ..................................................ggaaaaacccaaaugaacuu............................................ 1 0 seq
    ..................................................ggaaaaacccaaaugaacuuu........................................... 4 0 seq
    ..................................................ggaaaaacccGaaugaacuuu........................................... 1 1 seq
    ..................................................ggaaaaacccaaaugaacuuuA.......................................... 1 1 seq
    ...................................................gaaaaacccaGaugaacuuu........................................... 6 1 seq
    ...................................................gaaaaacccaGaugaacuuuu.......................................... 6 1 seq
    ....................................................aaaaacccaaaugaacuuu........................................... 1 0 seq
    ....................................................Gaaaacccaaaugaacuuu........................................... 1 1 seq
    ....................................................aaaaacccaGaugaacuuu........................................... 3 1 seq
    ....................................................aaaaaAccaaaugaacuuu........................................... 1 1 seq
    ....................................................aaaaacccGaaugaacuuuu.......................................... 2 1 seq
    ....................................................aaaaacccGaaugaacuuuuu......................................... 1 1 seq
    ....................................................aaaaacccaaaugaacuuuuu......................................... 15 0 seq
    ....................................................aaaaacccaGaugaacuuuuu......................................... 45 1 seq
    ....................................................aaaaacccaaaugaacuuuuA......................................... 1 1 seq
    ....................................................aaaaacUcaaaugaacuuuuug........................................ 12 1 seq
    ....................................................Gaaaacccaaaugaacuuuuug........................................ 14 1 seq
    ....................................................aaGaacccaaaugaacuuuuug........................................ 1 1 seq
    ....................................................aaaaacccaGaugaacuuuuug........................................ 2 1 seq
    ....................................................aaaaacccaaaugaacuuuuuUg....................................... 2 1 seq
    ....................................................aaaaacccaaaugaacuuuuugA....................................... 2 1 seq
    .....................................................aaaacccaaaugaacuuuu.......................................... 5 0 seq
    .....................................................aCaacccaaaugaacuuuuu......................................... 1 1 seq
    .....................................................aaaacccGaaugaacuuuuug........................................ 6 1 seq
    .....................................................aaaacccaaaugaacuuuuug........................................ 9 0 seq
    .....................................................aaaacccaaGugaacuuuuug........................................ 2 1 seq
    .....................................................aaaacccaaaugaacuuuuugA....................................... 1 1 seq
    .....................................................aaaacUcaaaugaacuuuuugg....................................... 3 1 seq
    .....................................................aaaacccaaaugaacuuuuuggA...................................... 1 1 seq
    ......................................................aaacccaaaugaacuuuuuggU...................................... 1 1 seq
    ......................................................aaacAcaaaugaacuuuuuggc...................................... 2 1 seq
    ...........................................................caaaugaacuuuuuggccaacc................................. 1 0 seq
```

```
                           Star                         Mature
uuuuguaagguauauugaauuggcccaauaguucauuugaguguaaauauggaaaaacccaaaugaacuuuuuggccaacccaguacauuaccuacucauacccacagucaug
............................................................aaaugaacuuuuuggccaacc................................   1   0   seq
............................................................aaaugaacuuuuuggccaacccaU.............................   1   1   seq
............................................................aaaugaacuuuuuggccaacccaUu............................   1   1   seq
..............................................................augaacuuuuuggccaacccaUu............................   1   1   seq
..............................................................augaacuuuuuggccaacccagA............................   1   1   seq
...............................................................ugaacuCuuuggccaacccagu............................   1   1   seq
```
